# Cloud-Assisted UAV Data Collection for Multiple Emerging Events in Distributed WSNs

**DOI:** 10.3390/s17081818

**Published:** 2017-08-07

**Authors:** Huiru Cao, Yongxin Liu, Xuejun Yue, Wenjian Zhu

**Affiliations:** 1 School of Electrical and Computer Engineering, Nanfang College of Sun Yat-sen University, Guangzhou 510970, China; caohr@mail.nfu.edu.cn (H.C.); zhuwj@mail.nfu.edu.cn (W.Z.); 2 College of Electronic Engineering, South China Agricultural University, Guangzhou 510642, China; liuyx@mail.nfu.edu.cn

**Keywords:** cloud-assisted, Emerging event, Flying parameters, UAV, WSN

## Abstract

In recent years, UAVs (Unmanned Aerial Vehicles) have been widely applied for data collection and image capture. Specifically, UAVs have been integrated with wireless sensor networks (WSNs) to create data collection platforms with high flexibility. However, most studies in this domain focus on system architecture and UAVs’ flight trajectory planning while event-related factors and other important issues are neglected. To address these challenges, we propose a cloud-assisted data gathering strategy for UAV-based WSN in the light of emerging events. We also provide a cloud-assisted approach for deriving UAV’s optimal flying and data acquisition sequence of a WSN cluster. We validate our approach through simulations and experiments. It has been proved that our methodology outperforms conventional approaches in terms of flying time, energy consumption, and integrity of data acquisition. We also conducted a real-world experiment using a UAV to collect data wirelessly from multiple clusters of sensor nodes for monitoring an emerging event, which are deployed in a farm. Compared against the traditional method, this proposed approach requires less than half the flying time and achieves almost perfect data integrity.

## 1. Introduction

With rapid advances of Information and Communication Technology (ICT), aerial robots, especially UAVs, have attracted the attention of both industry and academia. Because UAVs are flexible, portable, inexpensive, and convenient to use, they have been applied to perform various tasks in the domains of transportation, agriculture, healthcare, and heavy industry [1,2,3,4,5,6]. Certain characteristics such as being wireless, unmanned, and remotely-operable make them appropriate for use in domains which might be dangerous or difficult for humans. There have been several research studies for the application of UAVs for the protection of powers plants, inspection of electric power, disaster rescue, and recording aerial videos for the protection of the environment [7,8,9,10]. Traditionally, wireless sensor networks have been used and one drawback is lack of moving ability for fix-installed wireless nodes [11,12,13]. Although many studies have introduced mobile robots to address the problem, the locomotive capacity of these ground robots can often be limited by the terrain or obstacles of monitoring sites [14,15]. Therefore, more studies are integrating UAVs or other aerial robots into WSNs. These systems are called UAV-WSNs and they are well suited for remote surveillance and emergency response [16,17,18,19,20], especially for events such as landslides, wildfire, flood, etc. In these scenarios, real-time event surveillance is the basis of the right decision. Furthermore, to some extent, real-time event detection could save lives and cost.

However, UAV-based WSNs suffer from two major limitations: (a) due to the limited on-board computational capacity and storage, WSNs based on UAVs cannot perform global optimization algorithms for desired factors such as flying trajectory, and network topology control, (b) since WSNs and UAVs are powered by batteries [21], energy consumption is a major constraint, (c) there is a limited consideration of events priority. Therefore, we leveraged the cloud computing infrastructures to store and manipulate a large amount of data so as to undertake computational optimization for the behaviors of a WSN and UAV. Next, we provided a novel technique to minimize the energy utilized by the WSNs and UAVs to improve the duration of the data collection.

The rest of the paper is organized as follows: Section 2 discusses the related work on WSNs, data gathering using UAVs, current challenges and problems. In Section 3, an efficient architecture for data aggregation using a UAV-based WSN is presented. The problem description, the mathematical model and the algorithm based on the emerging event and user interests are given in Section 4. The goal in this section is to find the optimal flying parameters and the corresponding WSN nodes and clusters. In Section 5 we discuss the simulation results by comparing our method with other approaches. Finally, we provide our conclusions and discuss possible future work in Section 6.

## 2. Related Work and Contributions

UAVs have the ability to enhance the data collection system of WSNs with improved mobility, scalability, and flexibility. Therefore, in recent years, there has been a growing research interest in combining UAVs and WSNs for data gathering [22,23,24]. The related literature on the topic can be divided into two groups: (a) cloud-WSN, (b) applications of UAV-based WSN and (c) optimization of the parameters. In this paper, we focus on each of these three aspects, and they are our motivation for this paper.

**Cloud-WSN:** With the era of big data coming, the traditional WSN has faced many challenges, such as data storage, computing, etc., because of WSN’s limit abilities of storing and computing. So, in [25,26,27] and other works, researchers have begun to integrate WSN and Cloud for dealing with these new changes, and particularly the framework of sensor-cloud is proposed. In [27], the authors proposed a Multi-Method Data Delivery (MMDD) scheme for sensor-cloud users. This strategy incorporates four kinds of data delivery. Furthermore, in [28], to get good communication, the multiple mobile sinks (MSs) to help with WSN’S data collection are adopted in the literature, and a time adaptive schedule algorithm (TASA) for data collection via multiple MSs is designed, with several provable properties. In [11], the authors proposed a sensing service framework for integrating Wireless Sensor Networks and cloud infrastructure to impart climate alerts and perform climate analysis at reduced cost. It is obvious that WSN-Cloud or Sensor-Cloud is an effective way of dealing with the big data challenges; however, there are few works that focus on the WSN cloud applications and multiple emerging events.

**Application of UAV-WSN:** WSNs have been widely applied for parameter sensing in different environments, especially for deploying emergency applications in harsh conditions. In agricultural applications, UAV-WSNs have been used for collecting ground sensor data, surveillance crops, spraying chemicals, etc. In [16], the authors proposed a system for spraying chemicals on crops where the UAVs were responsible for spraying the chemicals, while the WSNs provided feedback and information from the ground. In [17], a system was developed for real-time frost surveillance in vineyards. A dynamic mobile node was carried by an UAV to ensure communication between the sparse clusters located at fragmented parcels and a base station. 

The flexibility of deploying UAVs have made them useful for surveillance special events. In [18], a UAV-based WSN was applied to surveillance wildfire in forests, while in [19], the authors, built a flight formation based on UAVs with WSNs and imaging systems. This system can fly over disaster areas such as wood fires or large traffic accidents. In these applications, for enhancing the scalability, and overcoming the drawback of the traditional WSN, UAVs play an essential role. They serve as the sink, sensing, relay nodes in the whole cloud-assisted UAV data collection system. However, these studies do not consider multiple events and the constrains of UAVs and hence lack the rigorous demands which practical applications would face. 

**Related optimizations:** Several researchers have studied the related optimizations of UAV-WSNs. In [29,30,31], to improve results, researchers experimented with optimization algorithms such particle swarm, ant colony, gradient descent to optimize the path planning of UAVs and WSNs. Other studies have focused on the communication protocol of the system. In [32], a new protocol, consisting of the TDMA and PFS schemes, was proposed to improve the reliability of the communication of WSN-UAV systems. In [23], the authors presented a data-centric routing protocol to support the establishment of a global gradient that only sends aggregated data from the center of the event to the data sink via multiple adjustable routes to increase the reliability. However, these works in the literature are normally based on quite specific assumptions, such as the fixed communication load, and the same priority of the sensing event. The current approaches have not considered the different parameters of WSNs, UAVs and other related constraints.

The contributions of this paper are listed as follows. We propose a cloud-assisted UAV data collection framework for distributed WSNs. The approach is based on the correlation between the area of emerging events and the WSN surveillance region. According to the event correlation matrix, the collected data clusters sequences are computed in the cloud and transmitted to the UAV. Meanwhile, based on global information including GPS position and data size of the ground WSNs, and the UAV basic parameters in terms of the flying height, position, and hovering time are calculated. The performance of our proposal is evaluated through simulation. To test our approach, we implemented a practical application in the real world.

## 3. Mathematical Model and Algorithm Analysis

In this section, to illustrate our approach to data collection using UAV, we present the outline of the algorithm and a mathematical model. We also provide the theoretical analysis of the algorithm.

### 3.1. Outline of Our Algorithm

**Figure 1:**
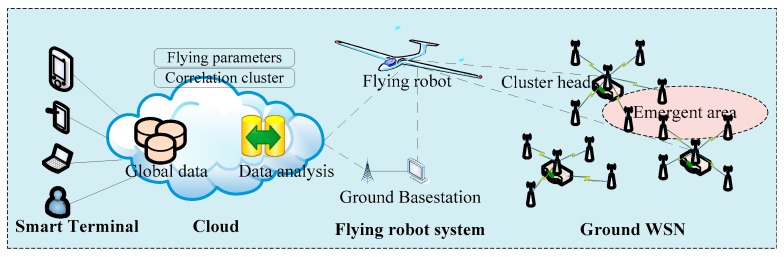
Topology of a hierarchical Unmanned Aerial Vehicles-Wireless Sensor Networks (UAV-WSN).

In the paper, we focus on emerging events surveillance in ground WSN, and use the UAV to enhance the data collection scheme. Figure 1 shows the schematic of the approach. The overall system includes a cloud computing platform, a UAV, sensor nodes and cluster heads. The process in our approach after the occurrence of specific events can be divided into two phases. The operation of the mechanism can be described by the following steps: cloud based mission planning and UAV data collection.

In the first step, if there are emerging events in the monitored area, the related and simple information is sent to the base station. Next, the cloud computing platform identifies the event-related clusters based on the position of the WSN clusters and the coverage of events. Meanwhile, the algorithm in the cloud is used to optimize the visiting sequence of clusters in the WSN. Then, the flight mission is scheduled to the UAV.

In the second phase, to save energy and shorten the flying time, optimal parameters for dynamic flying must be decided in advance. In this phase, issues such as rate of packet loss and data acquisition integrity of the wireless communication channel are addressed at the same time. 

### 3.2. Mathematical Definitions

To better illustrate the problem and describe the algorithm, we consider the characteristics of the WSN and the UAV along with actual applications. For this, we provide the following definitions and the assumptions made in the system:

(1) There are a limited number of ground WSN nodes in the clusters, and the network does not contain any mobile node.
(2) Within each cluster, there is only one cluster head. This node always has more energy than ordinary nodes.
(3) Each node has its own unique identification code and GPS position.
(4) The whole wireless network operates on a single fixed radio channel.
(5) The UAV cruises at the same speed during the entire process of data collection.

We consider that there are n clusters in the ground WSN. They are denoted by the set *C_N_* = {*c*_1_, *c*_2_, …, *c_i_*, …, *c_n_*}. Each cluster *c_i_* has *n_i_* sensor nodes, and each node *i* has the position *P_s_* (*x_i_*, *y_i_*). Furthermore, we assume that there are *m* emerging events, denoted by the set *U_M_* = {*u*_1_, *u*_2_, …, *u_j_*, …, *u_m_*}. Every event *u_j_* has an event radius of *R_u_* with central coordinates *P_u_* (*x_j_*, *y_j_*) and an event priority, denoted by *W_u_* = {*w*_1_, *w*_2_, …, *w_j_*, …, *w_m_*}. Based on the above assumptions, we have the following definitions:

**Definition 1.**  ***Event correlation coefficient:** Let $\alpha_{a}\left(j\right)$ be the total number of nodes in cluster a; and $\beta_{a}\left(j\right)$ be the number of nodes correlated with the event j. The event correlation coefficient of the cluster a, given by $\tau_{a}\left(j\right)$ is the ratio of the correlated and the total number of nodes in cluster a. This is given by Equation (1):*

(1) $$\tau_{a}(j)=\frac{\alpha_{a}(j)}{\beta_{a}(j)}$$

The correlation between the nodes and events is the foundation of our proposal. Let $\varphi \left(S_{i},U_{j}\right)$ denote the correlation between a node *S_i_* and an event *U_j_*. Let the sensing and event radii be *R_s_* and *R_u_*. Let *D* (*S_i_*, *U_j_*) be the distance from the node *S_i_* and the center of the event *U_j_*. $\varphi \left(S_{i},U_{j}\right)$ can have one of the two values: 1 or 0, which indicates whether *S_i_* and *U_j_* are correlated or uncorrelated, respectively. Therefore the $\varphi_{s}\left(i,j\right)$ of the node and the event is:

(2) $$\varphi (S_{i},U_{j})=\{\begin{array}{cc} 1, & \mathrm{if}\;D(S_{i},U_{j})\geq Rs+Ru\\ 0, & \mathrm{otherwise} \end{array}$$

We can derive the cluster correlation coefficient Φ(i,j) between the cluster *C_i_* and event *U_j_*, as:

(3) $$\underset{S\in C_{i}}{\Phi (i,j)}=\{\begin{array}{l} 1,\;\mathrm{if}\;\varphi_{s}>=1\\ 0,\;\mathrm{otherwize} \end{array}$$

Hence, we obtain the correlation matrix ${∆}^{m*n}$ between *Ci* and *U_M_*, where the rows and columns are the clusters and events, respectively. The ${∆}^{m*n}$ is mathematically written as follows:

(4) $$\Delta^{m*n}=\left(\begin{array}{ccc} \Phi_{11} & \ldots & \Phi_{1n}\\ \vdots & \ddots & \vdots \\ \Phi_{m1} & \cdots & \Phi_{mn} \end{array}\right)$$

**Figure 2:**
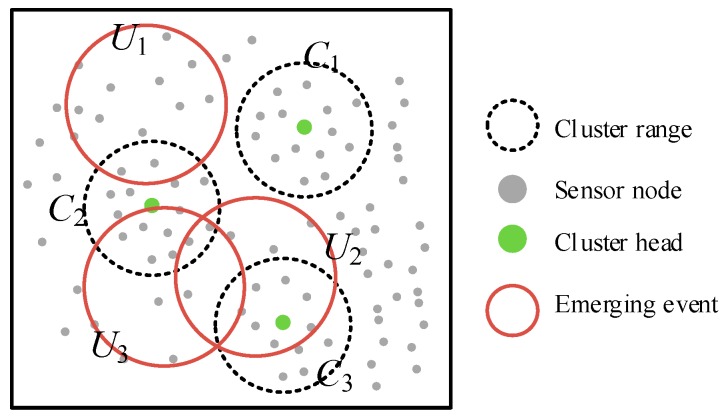
Modelling of Event correlation coefficient.

To better understand the essence of the event correlation coefficient matrix, let us consider an example shown in Figure 2 where the surveillance network consists of three sensor clusters (*C*_1_, *C*_2_, *C*_3_) and three events (*U*_1_, *U*_2_, *U*_3_) are trigging the alarm. We find that two sensors in cluster *C*_2_ could sense the event *U*_1_, so the cluster correlation coefficient for *C*_2_ and *U*_1_ $\Phi_{21}=1$. In the same way, the cluster correlation coefficient $\Phi_{11}=0$, as in cluster *C*_1_ no sensor could detect the event *U*_1_. So, the overall correlation matrix $\Delta^{3\times 3}=\left[\begin{array}{ccc} 0 & 0 & 0\\ 1 & 1 & 1\\ 0 & 1 & 1 \end{array}\right]$.

Let *Sc*(*f*) and *Sr*(*f*), be sums of the *f*th column and row respectively. From this, we can obtain the following Lemmas.

**Lemma 1.**  Given multiple events occurring in a cluster i, we have Sr(i) >1.

**Proof.**  For any cluster *i* which senses the event *j*, we have a cluster coefficient $\Phi_{ij}=1$. We assume that there are *L* = {1, 2, …, *l*} events occurring in the cluster *i*. Namely, ‖L‖=l>1. We can obtain the sum of the cluster correlation as $\Sigma_{j=1}^{l}\Phi_{ij}=\left\|L\right\|$. Since $L\subseteq U_{M}$, we get that the value of Sr(i)≥l>1.

**Definition 2.**  ***Integrity of data acquisition**: Let $\epsilon_{a}\left(j\right)$ and $\varepsilon_{a}\left(j\right)$ be the number of received and total messages, respectively, of the event j in the cluster a. ∂_a_ (j) is the ratio of $\epsilon_{a}\left(j\right)$ and $\varepsilon_{a}\left(j\right)$, namely:*

(5) $$\partial_{a}(j)=\frac{\in_{a}(j)}{\varepsilon_{a}(j)}$$

*So, the data acquisition integrity (ℓ(j)) of the event of j in WSN is given by:*

(6) $$\ell (j)=\frac{\Sigma_{a=1}^{n}\in_{a}(j)}{\Sigma_{a=1}^{n}\varepsilon_{a}(j)}$$

**Definition 3.**  ***Total Flying Score**: Let F_s_ be the total flying score. D_f_ is the total distance flown by the aerial vehicle to gather data for an event. So, at a given moment (t), F_s_ is the sum of the results of* $\alpha_{a}\left(j\right)$ *multiplied by the event priority and divided by the total flying distance. F_s_ can be written as:*

(7) $$Fs=\frac{\underset{a\in }{\Sigma }\underset{j\in M}{\Sigma }\alpha_{a}(j)\cdot w_{j}}{D_{f}}$$

### 3.3. Acquisition Sequence Design

For an event-driven UAV and WSN, every flight to collect cluster data must consider the flying distance, correlation and the event priority. In other words, we should strike a balance between these factors. The goal of this work is to maximize *Fs* of the WSN and can be formulated as:

(8) $$\begin{array}{l} \mathrm{Maximize}\;Fs\\ \mathrm{subject}\;\alpha_{a}(j),\beta_{a}(j),D_{f}>0 \end{array}$$

We formulate the problem of UAV collecting data from WSN for emerging events as a maximization problem. In this optimization process, the event correlation matrix plays a critical role. We divide the proposal into two stages. First, according to the distance, we obtain the hierarchal relation between the node and the cluster. Next, based on the total flying score, we select the correlation clusters for the UAV. A step-wise greedy strategy is used to deal with the problem. Algorithm 1 describes the rules for designing the path of an UAV for collecting a sequence of clusters from multiple emerging events. Algorithm 1 works as follows: Initially, *C_N_*, *U_m_*, *W_u_*, *R_S_* and *R_u_* are empty. Then, parameters values are inserted according to the sensors, clusters and the emerging events. It iteratively computes the node event correlation φ(s,i,j) and cluster event correlation Φ(i,j) using Equations (1)–(3) (see Steps 1–11). Then, from the Steps 13–18, the Total Flying Score *Fs for different solutions* are found according the Equations (4)–(7). Finally, the value of *Fs* is ordered from Max to Min. 

**Algorithm 1.** Based on the correlation of the flying sequence for multiple emerging events.**Input:** Cluster contains the Sensor *C_N_*, the emerging event *U_M_*, and the event priority *W_u_*, *R_s_*, *R_u_*.**Output:** Set of ordering correlation clusters *G* = {*g*_1_, *g*_2_, *g*_3_, …, *g_k_*}.1: **Begin**2:  **for**
*j* ← 1 to *m*
**do**3:   **for**
*i* ← 1 to *n*
**do**4:    **for**
*S* ← 1 to *C_i_* do *r*5:     **if**
*D*(*s*, *i*, *j* ) < *R_s_* + *R_u_*6:      φ(s,i,j) = 1, Φ(i,j) = 17:      $\alpha_{i}\left(j\right)\leftarrow \alpha_{i}\left(j\right)+1$8:     **end if**9:    **end for**10:   **end for**11:  **end for**12:  **for**
*i* ← 1 to *n*
**do**13:   **if**
Φ(i,j) > 114:    $\underset{a\in }{\Sigma }\underset{j\in M}{\Sigma }\alpha_{a}(j)\cdot w_{j}$15:   **end if**16:  **end for**17:  compute *F_s_* using Equation (7).18:  Ordering the *F_s_* from max to min19:**end**

### 3.4. Parameter Design for the UAV 

In Section 3.3, we focused on the problem of searching the correlation cluster for emerging events from the ground WSN. In this section, we deal with the parameter optimization of the UAV while considering the characters of ground WSN. This approach can be divided into the following steps: (1) the UAV obtains the correlation of the sequence of flying over the WSN clusters according to Algorithm 1. Then the UAV flies over the clusters and establishes wireless links; (2) the size of the cluster data stored by cluster head is first obtained, and the UAV computes the length of time to hover; (3) in the third step, the UAV and WSN clusters finish the data communication.

Meanwhile, for the UAV, we use *V_f_* to indicate the velocity of flying and *H* to indicate the height of flying. The set *G* = {*g*_1_, *g*_2_, *g*_3_, …, *g_k_*} indicates the ordering of the correlation clusters. The corresponding data set of the sizes of the cluster is given by *D* = {*d*_1_, *d*_2_, *d*_3_, …, *d_k_*}. Then, *T_s_* = {*t_1_*, *t_2_*, …, *t_k_*} denotes the set of stay times over the WSN cluster. It is easy to obtain the flying time *T*, using the following calculations:

(9) $$t_{i}=\frac{d_{i}}{V_{n}}$$

(10) $$T=T_{f}+\overset{k}{\underset{i=1}{\Sigma }}t_{i}$$

where, *T_f_* is flying time in seconds of the UAV and *V_n_* is the wireless transmission speed. We assume that the size of the data of every correlated node is a constant *D_c_*. From this, Equation (9), can be approximated as:

(11) $$t_{i}=\frac{\beta_{i}\cdot D_{c}}{\tau_{i}\cdot V_{n}}$$

Furthermore, we can derive the following properties: (1) the number of correlation clusters *K* is given by 0 < *k* <= *n*; (2) the stay time of the UAV over the cluster is inversely proportional to its *event correlation coefficient*. The details of the approach are given in Algorithm 2 which describes the rules for obtaining the dynamic flying parameters of the UAV in the presence of an emerging event. UAV flies over the WSN cluster according to the correlation clusters matrix *G*, and collect data wirelessly, as described in Steps 2–7. Meanwhile, from Steps 8–10, it uses the Equations (9) and (10) to compute the hovering time for UAV. Finally, UAV returns to its ground base station.

**Algorithm 2.** Compute the dynamic flying parameters and parameters for data transmission.**Input:** Set of correlation clusters *G* = {*g*_1_, *g*_2_, *g*_3_, …, *g_k_*}, *V_n_*, *V_f_*, *D_C_*.**Output:** Set of stay times over the WSN cluster *T_s_* = {*t_1_*, *t_2_*, …, *t_k_*}.1: **Begin**2:  **for**
*i* ← 1 to ‖G‖ = *k*
**do**3:   UAV flies over the WSN cluster *g_i_*4:   Create a wireless link with the cluster head of the cluster *g_i_*5:   Cluster head receives the correlation information from the sensor node6:   Send the *d*_i_ to UAV7:   UAV receives the *d*_i_, *D* ← *d*_i_8:   *t_i_ = d_i_/V_n_ // Compute the stay over cluster time*9:   *T_s_* ← *t_i_*10:    Hover *t_i_*, and Begin communication11:    Finish data transmission12:    Fly to the next cluster13:  **end for**14:  Fly to the ground base station15: **end**

## 4. Simulation and Results

Simulations were conducted to evaluate the performance and accuracy of the proposed algorithm. After analyzing the simulation results, the properties of the proposed approach were compared with that of the previous works discussed in related work. Comparisons were performed in terms of integrity of data acquisition, real time performance, length of flying time and energy consumption. The simulation parameters, setup and results are given in this section.

### 4.1. Simulation Setup

To measure the performance of our approach, a simulated UAV-WSN testbed for data collection was configured. The simulated environment is distributed in an area of 1000 m in length and 1000 m in width. We assume that (1) Each node has its own unique identification code and GPS coordinate; (2) The whole wireless network operates on a single fixed radio channel, (3) The UAV works at the same speed during the entire process of data collection; (4) The value sensing and event radius *Rs* and *Ru* are fixed. In total, 1000 sensors were randomly placed in the field and the whole network uses a data packet size of 100k bits. The emerging events occur randomly in the simulation area; the priority and the number of events is also randomly assigned. The flying altitude of UAV is 30 m, and its velocity is 5 m/s. The sensor node, the cluster head and the UAV communicate using the ZigBee protocol. Other parameters of the network are shown in Table 1. The simulation runs 100 times, and the evaluation indexes take the averages.

The virtual testbed is initialized as follows: First, the sensor nodes are randomly deployed on the simulation site. Then, using the k-means clustering algorithm, cluster heads are created. When the emerging events occur, the Algorithms 1 and 2 are applied. The parameters of the network are shown in Table 1.

### 4.2. Performance Comparisons

**Figure 3:**
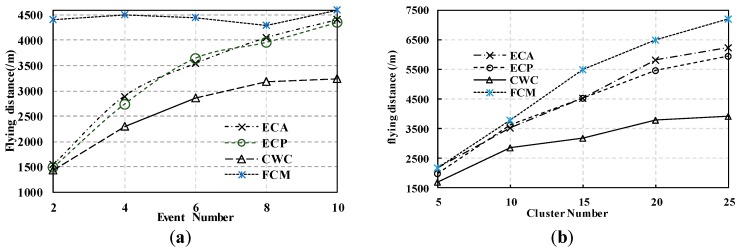
Comparison of the flight times and distances for UAV-sink. (**a**) Flight Time and Distances in different event number; (**b**) Flight Time and Distances in different cluster number.

***Flight Time and Distance***: To show the performance of the flight time and distance, we compare the results of our proposal with other methods: (1) the Full Collecting Method (FCM); (2) the event collection method which considers gathering all the data is called ECA; (3) the event collection method which considers the event priority is called ECP. Our approach, Cloud-assisted and Weight Event Data Collection, is called CWC. Figure 3 shows the assessment criteria of the flying time and the distance in Event-driven WSN (EDRW) using different methods. Experimenting with different numbers of events and clusters, we find that the flight distance of the CWC methods is much lower than the FCM, ECA, ECP methods. This is because, in the CWC method, calculating the shortest flying distance is one of the optimization objectives. In Figure 3a, we plot the results of 10 clusters with different event numbers. The flying distance is largest using the FCM approach. For ECA, ECP, and CWC, the flying distance gradually increases with the number of events. This is because the correlation cluster increases with the event number. Figure 3b plots the results of six event numbers with different cluster numbers. We see a similar trend in the flying distance for ECA, ECP, and CWC. In FCM, initially, the performance is better. However, the result gradually decreases and becomes worse after 10 clusters in EDRW. From the graphs, we can see that our approach outperforms the other strategies in terms of flying distance. Using our approach, a shorter flying time is used to collect event data from the ground WSN. Therefore, our proposal has the potential to reduce the energy consumption of the UAV.

**Figure 4:**
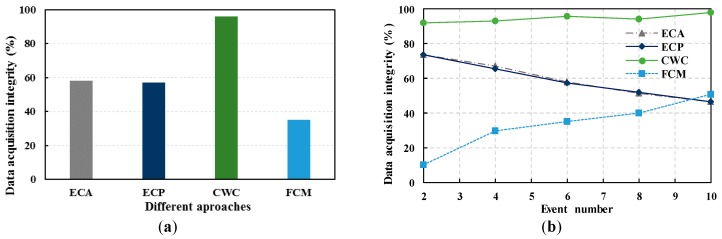
Comparison of Integration of Data Acquisition. (**a**) Integration of Data Acquisition with 10 clusters and 6 events in different approaches; (**b**) Integration of Data Acquisition in different event number.

***Integration of Data Acquisition:*** Figure 4 shows the converged data acquisition integrity of the wireless UAV in EDRW using the ECA, ECP, FCM, and CWC approaches. We can see that the data acquisition integrity in the CWC approach is higher than that in the ECA, ECP, and FCM approaches. The simulation results with 10 clusters and 6 events are shown in Figure 4a. The data acquisition integrity of the ECA, ECP, FCM and CWC approaches are 60%, 63%, 26%, and 97%, respectively. In the CWC approach, the UAV can stay over the WSN node for a time which is dependent on the data size, and gather information from the relevant nodes. In Figure 4b, the CWC has a high (greater than 92%) data acquisition integrity. In the ECA and ECP approaches, the values gradually decrease with an increase in the event number, as more sensor nodes and clusters become correlated with the events. Thus, an UAV must gather more data, which leads to the increasing in the amount of invalid information. However, the integrity of the data acquisition increases slightly, because with an increasing in the event number, FCM has a higher chance to collect valid data. From the graph, we can see that the CWC has a higher data acquisition integrity. Therefore, our proposal can gather more effective data for emerging events. Additionally, for the ground WSNs, the clusters would spend less energy and computational resources. This can prolong the work life of the WSN.

**Figure 5:**
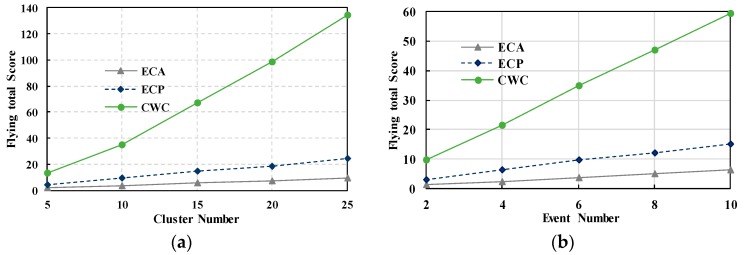
Comparison of Total Flying Scores. (**a**) Flying Scores in different cluster number; (**b**) Flying Scores in different event number.

***Total Flying Score:*** In Figure 5a, the total flying score of the ECA, ECP and CWC are plotted against different number of clusters. We can see that the CWC has a higher flying score than ECA and ECP. In the former, the ECA, ECP and CWC had slightly different scores when the numbers of clusters are low. However, with an increase in the number of clusters, the total flying score of CWC grows rapidly. This is because the CWC aims to optimize the flying distance, event priority, and cooperation with the ground WSN. Similarly, in Figure 5b, we can see the values and trends of the total flying score of the ECA, ECP, and CWC for different numbers of events. CWC has the best performance in this evaluation index. These values and the Figure 5 demonstrate that CWC approach is better than the other approaches for event monitoring. Therefore, we can state that our proposal is fit for data collection in emerging scenarios.

## 5. Real-World Use Case

In this section, a real-world use case is presented to further explain the benefits of our proposal in a light traffic UAV-WSN. For assessing the performance of CWC in real applications, we constructed the wireless network, UAV control system and carried out an experiment to compare our approach and other traditional schemes (FCM).

**Figure 6:**
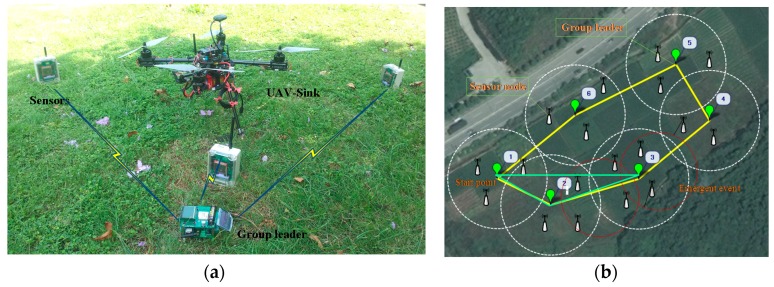
Multi-motor UAV sink. Right Flight path of UAV. (**a**) Implement scene; (**b**) UAV-Sink flying path in different ways. Note that, in Figure 6b, the yellow and green line indicate flying path using traditional means and our proposal, respectively. Red circles stand for emerging events.

Figure 6 shows a prototype platform of UAV-WSN for detecting the multiple emerging events, and this platform includes a variety of physical equipment, such as a UAV-sink node, sensor nodes, group leaders, etc. System configuration parameters are as follows. The antenna height of sensor nodes is 0.5 m; the transmission power is 1 dBm; the communication rate was 250 kbps; the node omni-directional antenna gain is 3 dBi. The wireless sensors are powered by batteries and equipped with CC2530 along with GPS module to get the position. WSN uses ZigBee as the wireless communication protocol, with a radio frequency of 2.4 GHz. The cluster heads are charged with photovoltaic panels. The UAV (a quadrotor) is controlled by an MCU stm32f103.

As mentioned above, sensor nodes are randomly placed in the surveillance area. After obtaining the communication quality index and its coordinate, the ground WSN is established. An auxiliary computer is connected to the WSN base station. As shown in Figure 6, the UAV followed this scheduled sequence of vesting and completes the WSN data collection accordingly. The Figure 6b also compares the flying path generated using traditional means (FMC) and our approach.

It is obvious that the cluster 3 could sense two emerging events, and the value of event correlation coefficient of cluster 3 is greater than the other clusters. So, UAV firstly collected the data of this cluster. After three rounds of experiments, we got the average assessment metrics of different criteria. The lengths flying time of CWC and traditional strategies are 5 min and 12 min respectively. Meanwhile, the collected data integrity is 99% and 45%, which is more than two times higher. Therefore, we draw the conclusion with confidence that the data communication quality between WSN and UAV is improved while the flying time and energy consumption of the whole cooperative system is obviously reduced. We make the system more efficient in terms of energy consumption and task response.

## 6. Conclusions

In this paper, we focus on the challenge of data collection using UAVs, optimizing the flying parameters such as altitude, flying trajectory, and the hovering time over the WSN. Based on the conditions of the ground WSN, which includes the quality of communication, and the positions of the cluster heads, we provided the related mathematical model and analyzed the properties of our method. We presented a cloud-assisted algorithm for data gathering from an emerging event. Moreover, a simulation is setup, and our proposal is verified in a real-world surveillance application. The results demonstrate that our strategy can reduce the flying time, distance, energy consumption and the latency of data collection. From the analysis of the experimental results, we show that our proposal outperforms the conventional methods.

The main constraint of this work is that we only considered the task of gathering event data in UAVs and WSNs using a single drone. In the future, we may need to consider extending our study to co-operate UAVs for more efficient data acquisition in large-scale emerging scenarios.

## Figures and Tables

**Table 1:** The UAV and WSN parameters.

Parameters	Values	Parameters	Valuess
*n*	5, 10, 15, 20, 25	*R_s_*	40 m
*m*	2, 4, 6, 8, 10	*R_u_*	40 m
*wj*	1~5	*H*	30 m
*V_n_*	250 kbps	*V_f_*	5 m/s
